# Epithelial cell migration requires the interaction between the vimentin and keratin intermediate filaments

**DOI:** 10.1038/srep24389

**Published:** 2016-04-13

**Authors:** Cristina Velez-delValle, Meytha Marsch-Moreno, Federico Castro-Muñozledo, Ivan J. Galván-Mendoza, Walid Kuri-Harcuch

**Affiliations:** 1 Department of Cell Biology, Center of Research and Advanced Studies, IPN. Apdo. Postal 14-740, México City, 07000, Mexico; 2 Confocal Microscopy Unit, Center of Research and Advanced Studies, IPN. Apdo. Postal 14-740, México City, 07000, Mexico

## Abstract

Epithelial migration plays a central role in development, wound repair and tumor metastasis, but the role of intermediate filament in this important event is unknown. We showed recently that vimentin coexists in the same cell with keratin-KRT14 at the leading edge of the migrating epidermal cells, and knockdown of vimentin impaired colony growth. Here we demonstrate that vimentin co-localizes and co-immunoprecipitates with keratin-KRT14, and mutations in the -YRKLLEGEE- sequence of vimentin significantly reduced migration of the keratinocytes. Our data demonstrates that keratinocyte migration requires the interaction between vimentin and keratins at the -YRKLLEGEE- sequence at the helical 2B domain of vimentin. These findings have broad implications for understanding the roles of vimentin intermediate filaments in normal and neoplastic epithelial cells.

Although vimentin (Vim) forms intermediate filaments in mesenchymal cells, it is also found in non-mesenchymal cell types such as epithelial cells and neurons during embryonic development^1^,^2^,^3^. It coexists with keratins in cultured keratinocytes^4^,^5^, in the outgrowths of epidermal explants *in vitro*^6^, and in some metastatic tumor cells, appearing as a possible marker of oncogenic progression^7^,^8^,^9^. In addition, in neo-natal hepatocytes undergoing epithelial-mesenchymal transition, Vim is co-expressed with keratins and formed a close association in the intermediate filament network, suggesting certain degree of overlapping^10^.

This has prompted the suggestion that Vim could reflect a phenotypic characteristic that contributes to the migratory and aggressive behavior of epithelial tumor cells^3^,^11^,^12^. However, despite the overwhelming amount of literature describing the above phenomena, the mechanism involved in the role of Vim in cell migration remains poorly elucidated. On the other hand, diploid keratinocytes also show Vim IFs in certain circumstances. We reported recently that a subpopulation of diploid Vim positive (Vim^+^) keratinocytes, involved in the growth of the keratinocyte colonies in human epidermal cultures, express the brightest α5β1 integrin and p63^13^ that have been considered putative stem cell markers in the epidermis^14^. In this paper we extended our understanding of Vim role in cell migration and we studied its mechanism of action using as a model system the diploid keratinocytes transfected with Vim mutated in the 2B domain.

## Results and Discussion

### Vim co-localizes with keratin-14 in human epidermal keratinocytes

**Figure 1:**
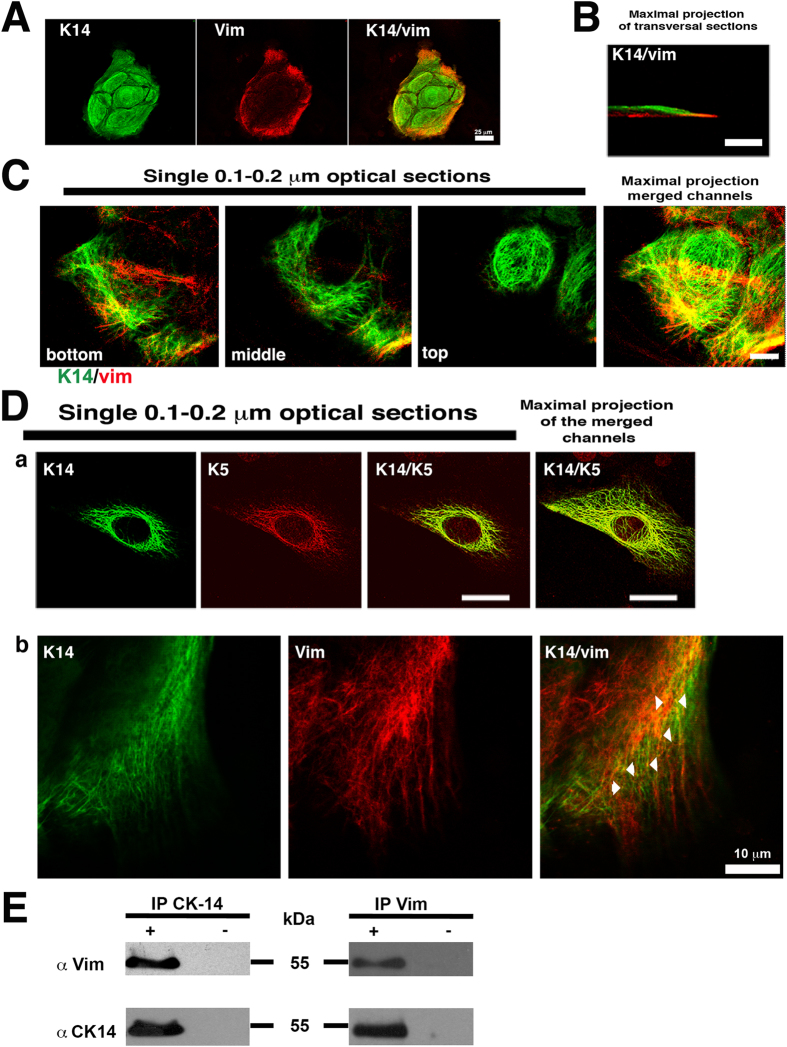
Vimentin and keratin stained in cultured human epidermal keratinocytes. (**A**) Immunofluorescence staining of a 4 day-old human keratinocyte colony for KRT14 keratin (green) and Vim (red). Note that only peripheral cells expressed Vim in addition to keratins. (**B**) Confocal microscopy image of a keratinocyte immunostained for KRT14 (green) and Vim (red). (**C**) Serial single optical sections by confocal microscopy showing the immunofluorescence staining for KRT14 and Vim at different focal planes. Note that while keratin IFs form a basket-like structure encasing the nucleus, Vim is present only in basal cells at the bottom. Scale bar = 10 μm. (**D**) Single optical section by confocal microscopy showing the immunofluorescence staining for KRT5/KRT6, or KRT14 and Vim; in (a) Note the complete co-localization of KRT14 with KRT5, and in (b) partial co-localization of KRT14 and Vim. (**E**) Representative immunoblot analysis showing the co-immunoprecipitation of Vim and KRT14, (+) in presence or (−) absence of the primary antibody.

To further study these Vim^+^ human epidermal keratinocytes, we used monoclonal antibodies for Vim, and for the basal cell keratins KRT5/KRT6 and KRT14. Some of the basal keratinocytes located at the periphery of the colony had Vim filaments, but stratified cells did not Fig. 1A; ref. 13. Vim filaments (IFs) were located mainly at the bottom of the cell below the nucleus and at the cell leading edge, which is in close contact with the tissue culture substrate (Fig. 1B), whereas keratin IFs formed a basket-like filament structure encasing the cell nucleus (Fig. 1C, top; Supplementary Video S1). This localization of the Vim IFs is similar to that found in fibroblasts in which long Vim IFs were seen extended from the nuclear region into the proximal region of the lamella, a structure mainly related to the motility of the cell^15^. Double staining of keratins KRT5 and KRT14, which co-polymerize, showed extensive co-localization, as expected (Fig. 1Da). However, at the migratory cell edge in some Vim^+^ keratinocytes, KRT14 and Vim showed co-localization at focal points (Fig. 1Db) with a Pearson’s correlation coefficient; Rr = 0.763 ± 0.110 and a Manders overlap coefficient; R = 0.957 ± 0.048. Both were measured in 57 optical sections samples (n = 57), as described by Zinchuk and Grossenbacher-Zinchuk^16^. Since Vim does not considerably copolymerize with keratin^17^, they are not likely to have extensive co-localization similarly to keratins KRT5 and KRT14. Since Vim and keratin can form staggered hybrid dimers and tetramers in copolymerization studies *in vitro*^17^, they must closely interact with each other intracellularly, as shown by our experiments. This interaction was confirmed by co-immunoprecipitation analysis (Fig. 1E); we found that Vim specifically co-immunoprecipated associated to KRT14 using either the MAb anti-KRT14 or the MAb against Vim (Fig. 1E).

### Knocking down the Vim gene disrupts keratinocyte migration

**Figure 2:**
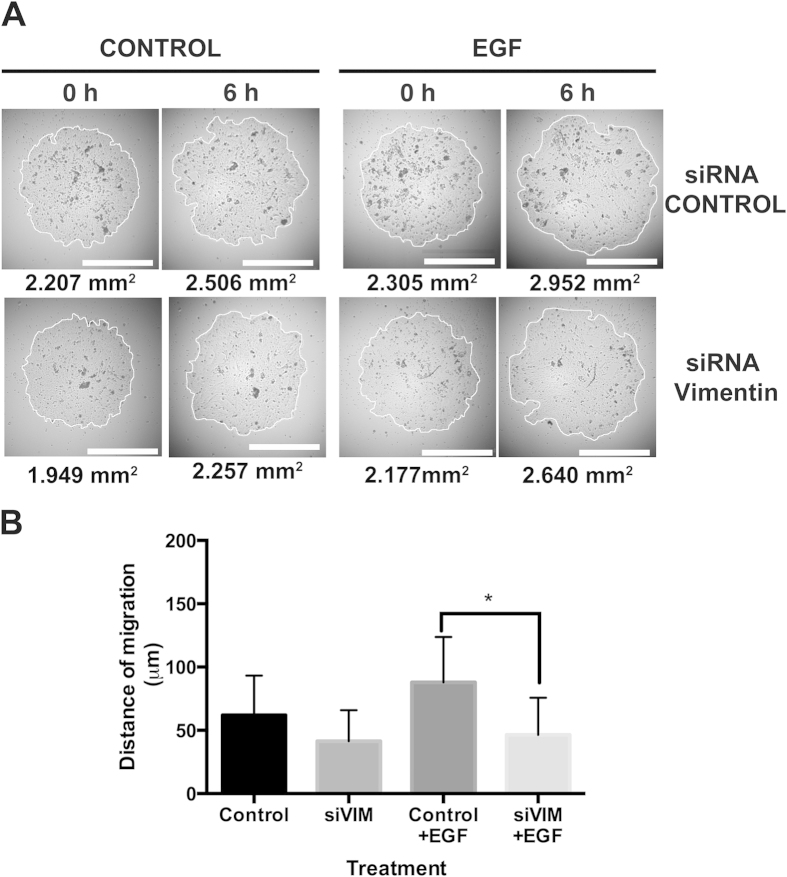
Silencing of Vimentin mRNA blocked the migration of cultured human epidermal keratinocytes. Keratinocytes were electroporated with a siRNA for Vim and they were seeded in a microliter radial monolayer assay (MRMA) described by Berens *et al*.^22^, which specifically measures cell migration. We seeded MRMA epidermal cultures for 12 h and thereafter treated them for 6 h with EGF, with or without the Vim siRNA. Then, after photographing the cultures under phase contrast microscopy, we determined the distance of migration by quantifying the area of the MRMA, and then calculated its radius. From these data, distance of migration was calculated. (**A**) Representative images of MRMA assay at 6 h. (**B**) Graph showing the distance of migration under each treatment. Note that cultures treated with siRNA for Vim had a reduced distance of migration in comparison to control cultures. Data is the mean of duplicate experiments with n = 16 each, ±SD *p ≤ 0.05.

We showed that Vim^+^ keratinocytes are mainly located at the periphery of the colony (Fig. 1A), and knocking down Vim mRNA, impaired keratinocyte colony growth^13^. Since the growth of a keratinocyte colony depends on the outward migration of the rapidly proliferating cells located in the thin rim of the colony perimeter^18^, and since Vim is involved in cell migration and invasiveness of carcinoma cells^19^, in order to study the role of Vim during diploid epidermal cell migration, we also knocked down Vim mRNA, and determined keratinocyte migration in a specific assay^20^,^21^, such as the microliter radial monolayer assay (MRMA) described by Berens *et al*.^22^, which specifically measures cell migration (see Materials and Methods). Silencing of the Vim gene caused 80% decrease in Vim mRNA at 48 hours post-transfection, and a concomitant reduction in its encoded protein, as we recently described^13^. We seeded MRMA epidermal cultures for 12 h and thereafter treated them for 6 h with EGF, with or without the Vim siRNA. Then, after photographing the cultures under phase contrast microscopy, we determined the distance of migration by quantifying the area of the MRMA (Fig. 2A), and then calculated its radius. Knocking down the Vim mRNA inhibited the migration of the keratinocytes (Fig. 2B). In the control MRMAs keratinocytes migrated 62 μm in 6 h, whereas in the EGF treated cultures they migrated 87 μm, and the Vim siRNA cultures treated with EGF migrated 49 μm, i.e. 21% less than in control cultures. The 87 μm of migration distance is equivalent to, at least, 7 cell diameters whereas that of control was 5, and that of the Vim siRNA cultures was 4 cell diameters. The cell diameter was considered to be about 12 μm as described previously^23^. The shortest doubling time of keratinocytes in exponentially growing microscopic colonies is about 17 h^23^. Our results also ruled out the possibility that migration was combined with cell proliferation in such a short time of treatment. These effects are much too rapid to be the result of increased proliferation in 6 h. If there were no cell migration, the MRMA would expand very slowly, since cell division alone could expand it by little more than 1 cell diameter per day. These results showed that knocking down Vim gene blocked keratinocyte migration.

### Vim-YRKLLEGEE-sequence interacts with keratin-14 to drive keratinocyte migration

**Figure 3:**
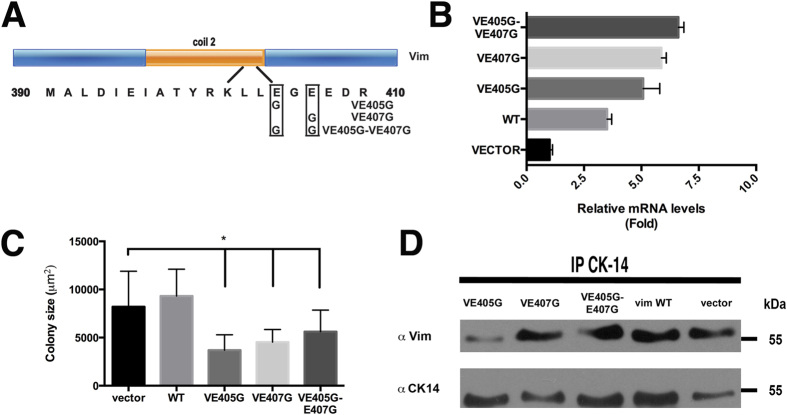
Vimentin binds to keratin via its YRKLLEGEE region located at the end of the coil 2B domain. (**A**) Schematic representation of the coil 2B domain of Vim showing the modified amino acids by site-directed mutagenesis. (**B**) Vim mRNA expression by qRT-PCR analysis from epidermal cultures that were transiently expressing Wt Vim or its mutants, at 48 h after transfection (**C**) Diameter of keratinocyte colonies from parallel cultures as in (**B**). Data is the mean of duplicate experiments with n = 16 ± SD *p ≤ 0.05. Note that mutated Vim decreased the size of keratinocyte colonies. (**D**) Immunoblot assays of the Vim proteins that were co-immunoprecipitated with KRT14 at 48 h post-transfection.

**Figure 4:**
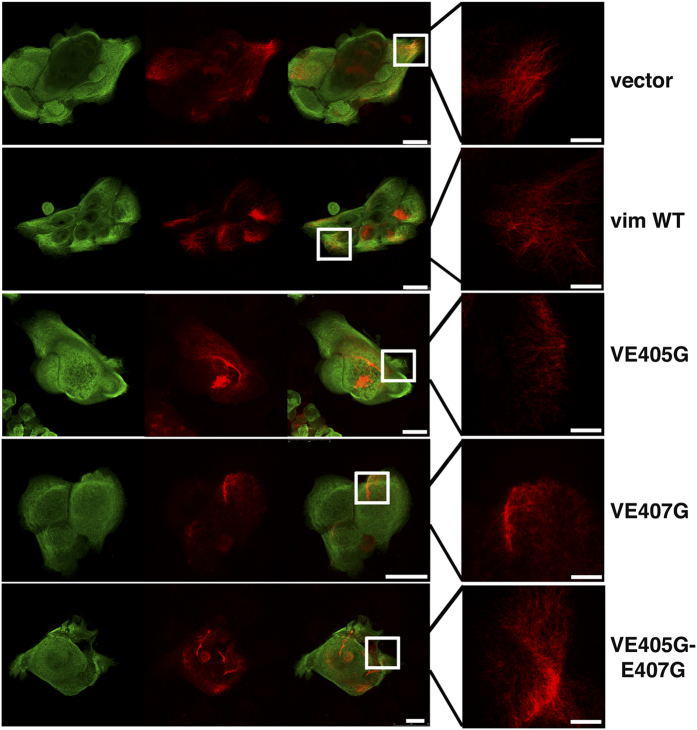
Mutations in the-YRKLLEGEE-region of Vimentin disrupts IFs arrangement. Representative images of the colonies measured in Fig. 3, showing disruption of Vim filaments. Scale bar = 25 μm; inset scale bar 7.5 μm.

The-YRKLLEGEE- sequence of Type-II keratins, such as KRT5, is required for interaction and co-polymerization with Type-I keratins, such as KRT14, that have a similar but not identical sequence (-YRRLLEGE-)^24^,^25^. The -YRKLLEGEE- sequence is essential to maintain the functional integrity of the keratin network, as a single mutation in KRT5 (-YRKLLGGEE-) can cause epidermolysis bullosa simplex^26^,^27^. Vim, at the end of the helical 2B-domain, has the same amino acid sequence -YRKLLEGEE- found in the type-II keratins^17^, and this sequence contributes to the IF dimer stability as it was reported in *in vitro* studies^25^. This raises the possibility that Vim could interact *in vivo* with KRT14 or other acidic keratins through such amino acid sequence. We tested this possibility by introducing point mutations by site-directed mutagenesis in the Vim sequence. We tested the mutation that occurred in KRT5 of epidermolysis bullosa by changing Glu 405 to Gly (V-E405G)^26^; a mutation in which the E407 was replaced by G (V-E407G); and a third mutation containing both modifications (V-E405G-E407G) (Fig. 3A). To our knowledge, these specific mutations have not been tested before in the Vim molecule. However, the deletion of the full coil 2B region of Vim in fibroblasts disrupts the cytoskeleton and delays apoptosis^28^. We carried out two types of experiments; one testing the colony expansion of growing keratinocytes, and two, the cell migration assay (MRMA) with 3T3 feeder cells (See Materials and Methods). By real-time PCR we showed a 2–6-fold increase in Vim mRNA in the transfected cells, in comparison with controls (Fig. 3B). We also found that all these three Vim mutants exerted a significant reduction in colony size (Fig. 3C) and, as it was expected, disrupted the Vim IFs (Fig. 4) since the -YRKLLEGEE- sequence contributes to the IF dimer stability^25^, and since we also showed the interaction between Vim with KRT14 (Fig. 1), it was also expected that the keratin filaments would be partially disrupted in the Vim^+^ keratinocytes (Fig. 4)), demonstrating that mutations in this Vim sequence inhibit colony expansion of the cells. The colony size of keratinocytes transfected with wild type Vim cDNA was similar to the controls, demonstrating that forced expression of Vim per se did not affect colony size (Fig. 3C). However, keratinocytes transfected with the mutated Vim genes showed about 50% smaller colony size (Fig. 3C).

**Figure 5:**
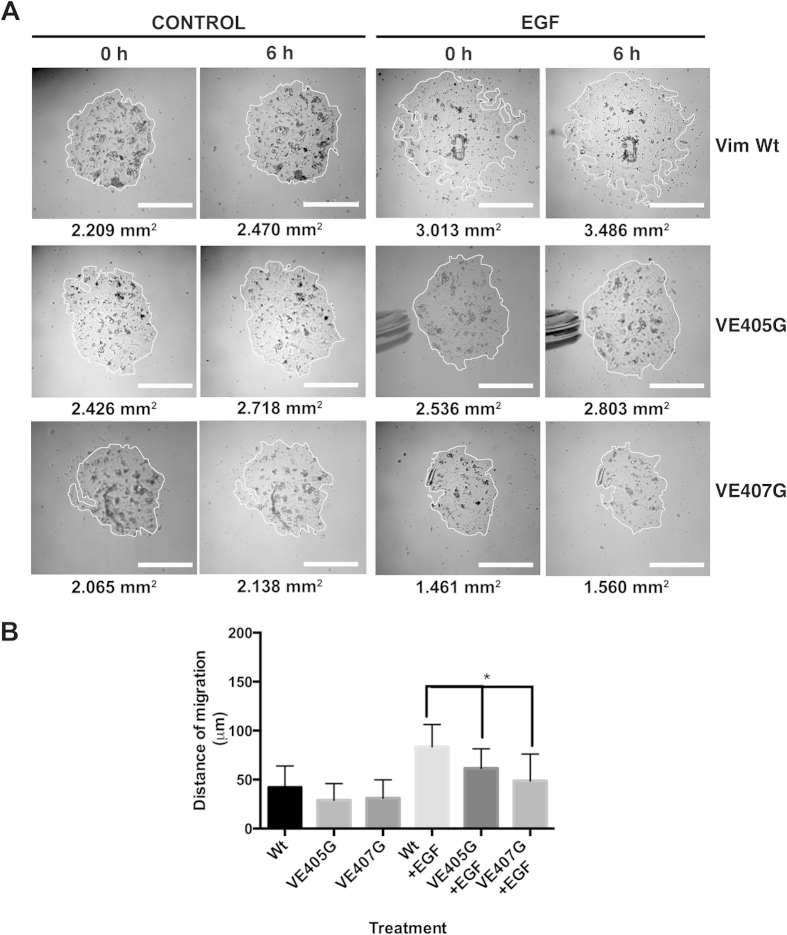
Decreased migration of keratinocytes electroporated with plasmid harboring Vimentin mutants in the –YRKLLEGEE- sequence of the Vimentin gene. Keratinocytes were electroporated with a Wt Vim or its mutants and they were seeded in a MRMA as above to measure cell migration. We seeded MRMA epidermal cultures for 24 h and thereafter treated them for 6 h with EGF, with or without the plasmid harboring the Vim genes. Then, after photographing the cultures under phase contrast microscopy, we determined the distance of migration by quantifying the area of the MRMA, and then calculated its radius. From these data, distance of migration was calculated. (**A**) Representative images of MRMA assay at 6 h. (**B**) Graph showing the distance of migration under each treatment. Note that cultures treated with the plasmid harboring the mutant Vim genes had a reduced distance of migration in comparison to control cultures. Data is the mean of duplicate experiments with n =16 each, ± SD *p ≤ 0.05.

Immunoprecipitation with the mAb against KRT14, V-E405G showed a lower content of immunoprecipitated Vim as compared to wild type, suggesting less association between the two molecules (Fig. 3D), whereas the other two mutants comprising amino acid 407 did not show significant changes in the content of the immunoprecipitated protein (Fig. 3D). Most importantly, we carried out functional experiments by the MRMA assay with the keratinocytes transfected with the plasmids harboring the mutants VE405G and VE407G (Fig. 5A), we found that each mutant reduced migration of the keratinocytes (Fig. 5B). In the MRMA control cultures without EGF cells migrated 34.2 μm and in the EGF treated cultures they migrated 78.1 μm, whereas in the V-E405G and VE407G transfected cultures and treated with EGF keratinocytes migrated 62.1 and 48.8 μm, 20 and 38% lower, respectively than in the control EGF stimulated cultures (Fig. 5B). Together, these results suggested that Vim interaction with keratins is necessary for cell migration, and more specifically, the -YRKLLEGEE- sequence seems to be a point of interaction between the two IFs in diploid keratinocytes.

IFs proteins have several points of interaction for heterodimerization^29^. For example, a similar point mutation in the –YRKLLEGEE- sequence of KRT5 promoted formation of keratin aggregates in PtK2 kidney epithelial cells, causing a grossly abnormal distribution of the keratin filament network involving an impaired heterodimerization between type-I and type-II keratins network^29^. We found an abnormal distribution of the KRT14 and Vim IFs in the keratinocyte transfected with the Vim mutants (Fig. 4). We can conclude that the -YRKLLEGEE- sequence is necessary for the integrity of the Vim IFs, and for Vim interaction with keratins to allow for cell migration. These results can be explained by data showing that Vim and KRT14 co-polymerized limitedly *in vitro*, forming staggered hybrid dimers and tetramers; but they were incapable of assembly into higher order structures; six peptides were likely to have formed between the Vim and KRT14 heterodimers^17^. Moreover, the addition of the Vim/KRT14 tetramer to an *in vitro* reaction mixture of KRT5/KRT14 assembly reaction, not only halted the further increase of the keratin IFs assembly but poisoned the reaction causing a disassembly of these IFs, as described by the same authors^17^. The formation of the staggered and limited hybrids seems to be explained by the different nearest neighbor alignments and different axial length of the molecules which is 43.9 nm for Vim and 46.2 nm for the keratin heterodimer making them incapable of co-assembly into higher order structures^17^. One of the polypeptides involved in the hybrid formation between Vim and KRT14 was that comprising the -YRKLLEGEE- sequence of Vim^17^. We could suggest that since the formation of intracellular hybrid Vim/KRT14, or more generally, Vim/type-I keratins, Vim would interfere through these structures in the interaction of type-I and type-II keratins. As it is shown by our mutagenesis experiments, these interfering would occur at least at the level of the -YRKLLEGEE- sequence, as part of the migration mechanism of epithelial cells. We cannot rule out other points of interaction between Vim and keratin IFs, but a similar effect would be expected. In epithelial cells a high content of keratin IFs gives additional mechanical strength and less flexibility to the cytoplasm. Since IFs are dynamic structures, we think it is conceivable that the ability of Vim to form the hybrid Vim/type-I keratin dimers or tetramers would disrupt at these focal points the rigidity of the type-I/type-II keratin filaments, facilitating the migratory ability of the epithelial cells. Indeed, our data shed light to this type of mechanism for epithelial cell migration. This interaction would explain previous results by electron microscopy in which immunogold co-localization between Vim and KRT18 (another type-I keratin) in neo-natal hepatocytes was found to be restricted to focal points in the filament network^10^. An additional observation we made is the encasing of the nucleus by a basket-like structure of the keratin IFs without Vim (Fig. 1C); this suggests to us that such a keratin cage should provide the rigidity required to pull the nucleus along the cell body contraction before the rear release during cell migration.

Epithelial-mesenchymal transition (EMT) is necessary for migration invasiveness of epithelial neoplastic cells^30^. EMT was initially described during embryogenesis converting epithelial cells into morphologically distinct mesenchymal-like cells without being irreversibly committed to this lineage^31^,^32^,^33^,^34^. Since, Vim gene expression and formation of Vim IFs are some of the important features in the EMT program in epithelial cells^30^, and since these cell types contains type-I and type-II keratins, we can hypothesize that the presence of Vim in the EMT would provide the basis of an interaction mechanism between Vim and type-I keratins to promote migration and invasiveness of the neoplastic cells.

Since Vim is part of the EMT in neoplastic cells^35^,^36^ it raises the possibility that targeting the EMT with drugs directed to disrupt the Vim IFs might provide a means for therapy of certain cancers. For example, the topical application of Vim siRNA, or withaferin-A that disrupts Vim IFs^37^, or substances interfering with the -YRKLLEGEE- sequence interaction, might be an effective therapy in controlling the growth of squamous cell carcinomas of the skin. It was reported that silencing of the Vim gene in breast carcinoma cells decreases their invasive properties by inhibiting the elongation of invadopodia^38^. More specifically, 2B2, a dominant-negative mimetic peptide derived from the C-terminal end of the central rod domain (residues 355–412 contain the -YRKLLEGEE- sequence) has been shown to inhibit the assembly of Vim IFs *in vitro* in fibroblastic cells^39^, and it results in the appearance of short filaments alongside the remaining long Vim IFs^15^. It is possible that a dominant-negative mimetic peptide derived from the -YRKLLEGEE- sequence might also disrupt the possible interaction between the Vim and Keratin IFs in the epithelial cells, serving as a more specific topical therapy for the skin carcinoma. Squamous cell carcinoma is characterized by the increased proliferation of basal keratinocytes containing Vim IFs that will facilitate migration of those basal keratinocytes and their invasion into the dermal compartment. It is well known that Vim is expressed in highly migratory and neoplastic epithelial cells, and since neoplastic cells show a similar association of Vim and keratins, we can conclude that a mechanism comprising direct interaction between Vim and keratin molecules at the -YRKLLEGEE- sequence takes place not only for the migration of normal epithelial cells, but also for the migration and invasiveness of epithelial neoplastic cells, such as in carcinomas or mesotheliomas, which also have a high content of Vim and keratins.

Furthermore, neural development involves a transition from mitotic, motile cells into post-mitotic, non-motile cells during which Vim is found initially in almost all neural precursors, declining shortly after cell division ceases. Expression of *Vim* increases transiently during axogenesis^40^, and its silencing causes inhibition of axonal neurite formation^41^; also, Vim is upregulated in the axoplasm after nerve injury^42^. Moreover, re-expression of the Vim gene after its expression declines during normal development induced a rapid elongation rate in axonal neurites even beyond the time when their outgrowth would normally have slowed or stopped^43^. During axonal growth, the neurofilament proteins, H, M and L co-assemble transiently with Vim into filamentous structures^44^,^45^. The protein blast analysis of the H, M and L proteins that form the intermediate filaments of nerve cells show similar structural features including the -YRKLLEGEE- sequence which is alike to the-YRRLLEGE- sequence of KRT14^46^. Therefore, we can hypothesize that Vim interaction with H, M or L proteins might occur during neurite and axonal growth and migration, similarly to what we have observed in the epidermal cells with keratins. Our study provides new understanding in the mechanism of action of Vim to facilitate epithelial cell migration through a new concept, raising a significant role for the interaction of Vim with keratin IFs, which it has been unknown to occur in cells during a physiological process such as cell migration. If this is true, the mechanism we are describing that involves Vim interaction with other intermediate filament proteins should have a broad implication in epithelial and neurite biology.

## Materials and Methods

### Materials

Insulin, hydrocortisone, BSA, human transferrin, cholera toxin, L-triiodothyronine, and mitomycin-C were purchased from Sigma Chemical Co. (St. Louis, MO). FBS was from HyClone Laboratories (Logan, UT). EGF was from Upstate Inc., (Charlottesville, VA). The antibodies used are described in Table 1. All other reagents were analytical grade.

### Human tissue samples

The human epidermal keratinocytes, strain HE-123, were isolated in our laboratory in 1991 from newborn foreskins obtained under protocols approved by the internal review board from Hospital MIG, Mexico, after parental written informed consent. All experiments were performed according to the Declaration of Helsinki protocols and were approved by the internal ethical committee of the Centro de Investigacion y Estudios Avanzados.

### Cultivation of human epidermal keratinocytes

The human epidermal keratinocytes (primary cultures and strains HE-54, HE-123, and HE-132) were isolated from newborn foreskins and cultured as described previously^47^. The 3T3 J fibroblasts were a generous gift from Dr. H. Green (Harvard Medical School), and they were cultured and treated with a lethal dose of mitomycin-C for use as feeder cell layers as previously described^48^. Single-cell suspensions of keratinocytes were plated at 2.7 × 10^3^ cells/cm^2^, together with 2.2 × 10^4^ cells/cm^2^ feeder cells using a (3:1) DMEM-F12 nutrient mixture supplemented with FBS, plus 5 μg/ml insulin, 0.4 μg/ml hydrocortisone, 2 × 10^−9^ M L-T^3^, and 24.3 mg/l adenine. Two days after plating, cultures were changed to medium containing 10 ng/ml EGF. In all experiments, media were changed every 3 days, and the cultures were maintained at 37 °C in a 10% CO_2_ and 90% air humidified atmosphere.

### Keratinocyte migration assay

The cell migration assay was carried out as described by Berens *et al*.^22^ using a microliter radial monolayer assay, with modifications. Transfected keratinocytes were seeded in 35-mm tissue culture dishes at 4500 cells in 1 microliter forming a 2 mm diameter drop to form a circular confluent monolayer. At 12 or 24 h after seeding as described in the experiments, 1 ml medium was added and a photograph was taken under the microscope to draw the edges of the circular confluent monolayer circumscribing the cells in order to measure the area at time zero. The medium contained 10 μg/ml EGF to stimulate migration according to Barrandon and Green^18^, with or without the siRNA for Vim, or the Vim mutants. Afterwards photographs were taken at 6 h, and the area was measured again as above. To determine the distance of migration the drawn surfaces were fitted to the best circle and radius was calculated. Distance of migration corresponds to the difference between both calculated radiuses. For the assays with the Vim mutants, we seeded a rim of feeder cells using an elastomer mask^49^; after removing the elastomer we carried out the MRMA as described above.

### Immunofluorescence staining

Cells were grown on 18.0 × 18.0-mm glass cover slips and fixed and permeabilized with ice-cold methanol-acetone (1:1) for 5 min. Afterwards, immunodetection was carried out as previously described^13^; fluorochrome-labeled primary antibodies were used. Slides were washed with PBS and mounted in Dako fluorescent mounting medium (Dako Corporation, CA). Confocal analysis was performed using a Leica high-speed SP5 tandem two-photon system (Leica Microsystems, Germany), equipped with a 63 NA 1.2 objective. For co-localization experiments, xyz and xzy serial optical sections 0.2 μm were captured. To prevent interference from the fluorescent probes, images of the same optical sections were captured as separate channels between frames, and they were analyzed with LAS AF v.1.8.0 (Leica Microsystems, Germany) according to acquisition of the images. All observations were carried out from at least three independent cell experiments. For colony size assay, transfected cells were seeded along with feeder cells; after 48 h they were fixed and immunostained using a Mab against CK14-fluorescein conjugate; 50 colonies were measured from each treatment. The photographs were reduced to printing size with Adobe Photoshop v.7.0 software without changing the number of pixels.

### Immunoprecipitation

Cultures of human epidermal keratinocytes were extracted with a buffer of 10 mM Tris-HCl (pH 7.4) containing 100 mM NaCl, 1.0 % Triton X-100, 0.1% SDS, 0.5 % sodium deoxycholate, 1.0 mM EDTA, 1.0 mM EGTA, 1.0 mM of NaF, 2 mM NaOV, 20 mM Na_4_P_2_O_7_, 1 mM PMSF, and Complete Protease Inhibitor Cocktail (Roche Diagnostics). Immunoprecipitation was carried out with monoclonal antibody against Vim or with a monoclonal antibody against KRT14, and a negative control without the primary antibody, in an overnight incubation with Dynabeads® protein G (Invitrogen, CA). Immunoprecipitated proteins were recovered by denaturing elution in Laemmli buffer containing 150 mM dithiothreitol, analyzed by SDS-polyacrylamide gel electrophoresis and detected by immunoblot as described above. Each experiments was carried out with two independent cultures by duplicate.

### Expression vector and mutagenesis

Site-directed mutagenesis was carried-out on plasmid DNA containing the Ultimate™ ORF clone of Vim acquired from Invitrogen. The mutations were inserted using dsDNA and PCR as previously described^50^. The primers harboring the mutant were for the mutant VE405G a change of Glu 405 to Gly 5′-AGGAAGCTGCTGG**G**AGGCGAGGAGAGC-3′, for the mutant VE407G 5′-GAAGCTGCTGGAAGGCG**GA**GAGAGCAGGATTTCTCT-3′ and for the modification including the two amino acids change VE405, 407G mutant 5′-CTACAGGAAGCTGCTGG**G**AGGCG**GA**GAGAGCAGGATTTCTCT-3′ (bases in bold in the primer sequences indicate the changes introduced in the DNA sequences). The introduction of the mutations were verified by endonuclease digestion for the addition or suppression of two different restriction sites. The wild type or mutant Vim were subcloned to pcDNA 6.2/V5-DEST GW vector, using the Gateway® (Invitrogen) recombination cloning technology according to the manufacturer specifications. The analysis of Vim mRNA expression by qRT-PCR was carried out as previously described^13^.

### Synthetic siRNAs

Synthetic siRNA against mRNA encoding Vim and scrambled siRNA were obtained from Ambion, Inc. The scrambled sequence Silencer Negative Control 2 RNAi was used as a negative control.

### Transfections

HEK were transiently transfected by electroporation with an Amaxa nucleofector under the program T-24, according to specifications recommended by the manufacturer, 1 × 10^6^ cells were used in each assay and transfection efficiency was evaluated in parallel cultures for the expression of GFP.

### Statistical analysis

Quantitative results are the mean, +/−standard deviation, of two or three independent experiments by triplicate. Qualitative data corresponds to one representative experiment by duplicate. Data were analysed by Student’s t-test and statistical differences were set when *P* value was lower than 0.05.

## Additional Information

**How to cite this article**: Velez-delValle, C. *et al*. Epithelial cell migration requires the interaction between the vimentin and keratin intermediate filaments. *Sci. Rep*. **6**, 24389; doi: 10.1038/srep24389 (2016).

## Supplementary Material

Supplementary Information

Supplementary Video S1

## Figures and Tables

**Table 1:** Antibodies used.

Antigen	Antibody specificity	Source
Vim	Monoclonal (clone V9), against human, rat, pig or avian; non cross-reactive with mouse; for immunostaining rhodamine conjugated	Santa Cruz Biotechnology Inc. (Santa Cruz CA)
CK K14	Monoclonal (clone L001), reactive with mouse, rat, human, porcine and canine	Santa Cruz Biotechnology Inc. (Santa Cruz CA)
	Monoclonal (clone L002), reactive with human; fluorescein conjugated	EMD Millipore (Billerica, MA)
CK K5/6	Monoclonal, clone D5/16 B4	Chemicon International, Inc. (Temecula, CA)
