# Supplementary material for: Epithelial cell migration requires the interaction between the vimentin and keratin intermediate filaments
Source: Sci Rep. 2016 Apr 13;6:24389. doi: 10.1038/srep24389 (PMC4829867; doi:10.1038/srep24389)
Supplement: Supplementary Information [file srep24389-s1.doc]

Supplementary Information from:

**Epithelial cell migration requires the interaction between the vimentin and keratin intermediate filaments**

Cristina Velez-delValle^1^, Meytha Marsch-Moreno^1^, Federico Castro-Muñozledo^1^, Ivan J. Galván-Mendoza^2^ and Walid-Kuri-Harcuch^1*^.

^1^Department of Cell Biology, ^2^Confocal Microscopy Unit, Center of Research and Advanced Studies – IPN. Apdo. Postal 14-740, México City, 07000. MEXICO.


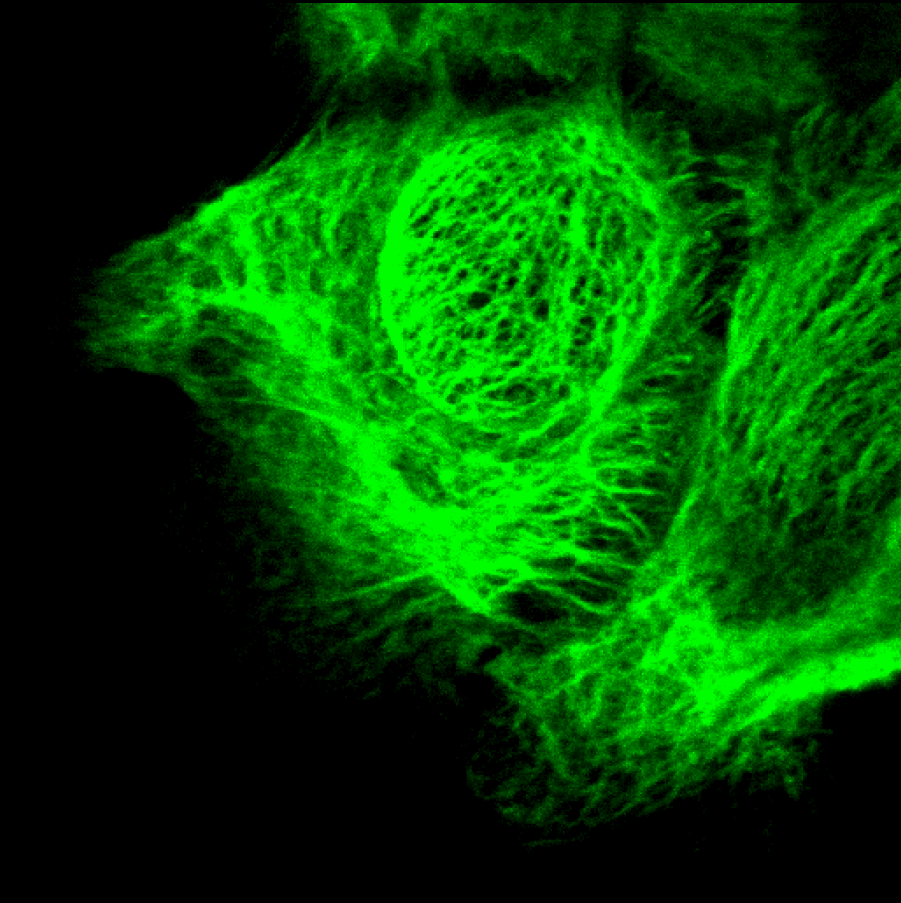


**S1 Video Description**. Tridimensional reconstruction of the keratin-KRT14 IFs, showing a basket-like filament structure encasing the cell nucleus.
